# Special Issue “Molecular Underpinnings of Schizophrenia Spectrum Disorders”

**DOI:** 10.3390/ijms27010188

**Published:** 2025-12-24

**Authors:** Claudio Brasso, Paola Rocca

**Affiliations:** Department of Neuroscience ‘Rita Levi Montalcini’, University of Turin, 10126 Turin, Italy

Schizophrenia spectrum disorders are complex, highly heritable mental conditions, with heritability estimates around 80–83%, affecting approximately 0.5–1% of the adult global population [1,2]. Characterized by diverse symptoms including disturbances in cognition, perception, affect, and behavior, their etiology is multifactorial, involving an intricate interplay of genetic, environmental, and neurobiological factors [2,3]. These disorders are often associated with significant functional impairment and disability, impacting daily living, social integration, and occupational capabilities [4,5]. Recent advancements in ‘omics’ technologies, such as genomics and transcriptomics, and innovative research methodologies, including induced pluripotent stem cells, are significantly enhancing our understanding of these disorders [6,7].

This Special Issue set out to explore the multifaceted molecular landscape of schizophrenia, seeking to integrate diverse research strands into a more cohesive understanding of its etiology, pathophysiology, and potential therapeutic targets. The contributions highlighted herein collectively advance this goal by shedding light on genetic predispositions, neuroinflammatory processes, the regulatory roles of non-coding RNAs, innovative biomarker discovery methods, and insights into cognitive function and suicide risk.

*Genetic and Genomic Contributions*

Baroni et al. [8] significantly deepened our understanding by proposing a distinct neurobiology for treatment-resistant schizophrenia (TRS). It suggests that TRS is characterized by a higher burden of specific genetic variants, including metabolic polymorphisms and glutamatergic/GABAergic gene variants, as well as an increased load of polygenic risk scores and copy number variants [8]. This differentiation from treatment-responsive forms highlights the necessity for tailored genetic investigations and treatment strategies for TRS. Furthermore, Iannotta et al. [9] provide compelling evidence, through a case study, that many schizophrenia-associated genetic variants exhibit pleiotropy, leading to shared genetic underpinnings with other neurodevelopmental disorders. This supports the concept of a neurodevelopmental continuum, emphasizing how a cumulative impact of inherited and de novo mutations can influence neurobiological development. Complementing this, Trombka & Meiron [10] bridge the gap between genetic predisposition and functional brain anomalies. It explicitly links high-risk genetic factors like GRIN2A and AKAP11 to specific disruptions in neural signaling, synaptic plasticity, and circuit function, thereby elucidating the mechanistic basis by which genomic alterations contribute to the “disconnection hypothesis” in schizophrenia.

*Neuroinflammation and Oxidative Stress*

Lv et al. [11] offer a comprehensive overview of the role of inflammatory cytokines in schizophrenia. This review consolidates findings that underscore chronic central nervous system inflammation as a key pathogenic factor, detailing how dysregulated pro-inflammatory cytokines can disrupt neurotransmitter balance, induce oxidative stress, and cause neuronal damage, thereby accelerating disease progression [11]. It also identifies specific cytokines (e.g., sIL-2R, IL-3, CCL11) whose altered levels in schizophrenia highlight their potential as diagnostic and prognostic biomarkers. Further refining this perspective, Baroni et al. [8] specifically distinguish neuroinflammatory signatures in treatment-resistant schizophrenia. This research reveals that TRS is associated with increased expression of inflammasome proteins in brain glial cells (microglia, astrocytes, oligodendrocytes), indicating more profound immune dysregulation and synaptic interference in these patients [8]. The study also strengthens the link between TRS and heightened oxidative stress, alongside reduced glutathione levels, providing distinct targets for intervention in this challenging subgroup.

*The Role of Non-Coding RNAs (ncRNAs)*

The scoping review by Li et al. [12] significantly consolidates evidence on the critical role of microRNAs (miRNAs) in schizophrenia pathogenesis. It elucidates how miRNA dysregulation impacts crucial neurological pathways, including those governing dopamine, glutamate, and immune responses, thereby positioning miRNAs as promising diagnostic and prognostic biomarkers [12]. Extending this focus to a broader class of regulatory molecules, Zhu et al. [13] provide a comprehensive analysis of long non-coding RNAs (lncRNAs). This systematic review highlights that lncRNAs are not only significantly altered in psychiatric disorders like schizophrenia but also hold substantial potential as highly sensitive and specific biomarkers for early detection, diagnosis, and even as novel therapeutic targets. This introduces a burgeoning area of research that complements the established understanding of ncRNAs.

*Biomarker Discovery and Innovative Methodologies*

Rubio-Contreras et al. [14] introduce hair matrix analysis as an innovative and less invasive methodology for biomarker discovery. This article underscores the untapped potential of hair samples for measuring crucial indicators such as neurotransmitter levels and epigenetic modifications, thereby suggesting a novel research avenue that could overcome limitations of traditional biofluid sampling. Furthermore, Farkas et al. [15] showcase the transformative power of human-induced pluripotent stem cell (hiPSC) technology in schizophrenia research. This work demonstrates how hiPSC models, particularly those derived from patients with specific genetic mutations like ZMYND11, can effectively replicate aspects of abnormal neural development and enable in vitro testing of antipsychotic drug responses. This represents a significant methodological leap in modeling the complex neurobiological aspects of the disorder and evaluating therapeutic interventions.

*Cognitive Function and Suicide Risk*

Paribello et al. [16] provide a nuanced perspective on suicide risk in psychotic disorders. This study reveals that individuals with a history of suicide ideation and attempts often exhibit a characteristic profile, characterized by lower negative symptoms and better performance in specific cognitive tasks [16]. This finding challenges conventional assumptions and highlights the importance of comprehensive neuropsychological profiling over mere categorical diagnoses for identifying individuals at higher risk for suicidal behaviors.

The current research landscape, illuminated by the studies presented, emphasizes the multifaceted nature of schizophrenia, driven by an intricate interplay of genetic vulnerability, neuroinflammatory processes, and molecular dysregulations. Significant progress has been made, and the integration of advanced biomolecular technologies and sophisticated analytical methods is paving the way for a deeper understanding of its etiology and progression, along with the identification of potential biomarkers and therapeutic targets. Future efforts should focus on validating these identified biomarkers through larger, longitudinal studies across diverse populations, which is crucial for their clinical translation.
